# Attainment rate as a surrogate indicator of the intervertebral neutral zone length in lateral bending: an in vitro proof of concept study

**DOI:** 10.1186/s12998-015-0073-8

**Published:** 2015-10-01

**Authors:** Alexander C Breen, Mihai Dupac, Neil Osborne

**Affiliations:** School of Design Engineering and Computing, Bournemouth University, Bournemouth, BH1 5BB UK; Anglo-European College of Chiropractic, Bournemouth, BH5 2DF UK

## Abstract

**Background:**

Lumbar segmental instability is often considered to be a cause of chronic low back pain. However, defining its measurement has been largely limited to laboratory studies. These have characterised segmental stability as the intrinsic resistance of spine specimens to initial bending moments by quantifying the dynamic neutral zone. However these measurements have been impossible to obtain in vivo without invasive procedures, preventing the assessment of intervertebral stability in patients. Quantitative fluoroscopy (QF), measures the initial velocity of the attainment of intervertebral rotational motion in patients, which may to some extent be representative of the dynamic neutral zone. This study sought to explore the possible relationship between the dynamic neutral zone and intervertebral rotational attainment rate as measured with (QF) in an in vitro preparation. The purpose was to find out if further work into this concept is worth pursuing.

**Method:**

This study used passive recumbent QF in a multi-segmental porcine model. This assessed the intrinsic intervertebral responses to a minimal coronal plane bending moment as measured with a digital force guage. Bending moments about each intervertebral joint were calculated and correlated with the rate at which global motion was attained at each intervertebral segment in the first 10° of global motion where the intervertebral joint was rotating.

**Results:**

Unlike previous studies of single segment specimens, a neutral zone was found to exist during lateral bending. The initial attainment rates for left and right lateral flexion were comparable to previously published in vivo values for healthy controls. Substantial and highly significant levels of correlation between initial attainment rate and neutral zone were found for left (Rho = 0.75, *P* = 0.0002) and combined left-right bending (Rho = 0.72, *P* = 0.0001) and moderate ones for right alone (Rho = 0.55, *P* = 0.0012).

**Conclusions:**

This study found good correlation between the initial intervertebral attainment rate and the dynamic neutral zone, thereby opening the possibility to detect segmental instability from clinical studies. However the results must be treated with caution. Further studies with multiple specimens and adding sagittal plane motion are warranted.

## Background

Low back pain (LBP) is a growing problem which is responsible for major population disability [1]. In the absence of a specific pathological or neurological cause, most LBP is classified as ‘non-specific’ and is often assumed to be mechanical if the pain is made better or worse by movement or position [2–4]. Lumbar segmental instability is thought to be an important factor in this, but for which there is no single definition or clinically available method for detection in patients [5, 6]. However, a generally accepted definition of clinical instability is “loss of the normal pattern of spinal motion causing pain and/or neurologic dysfunction” [7]. Many laboratory studies have explored this in terms of the neutral zone (NZ), which is the size of the zone of displacement when the bending moment is minimal [8, 9]. This measure has been found to be a more sensitive motion parameter in defining the onset and progression of spinal injury than the elastic zone or range of motion [10]. However, the measurement of the NZ has traditionally been impossible to obtain in vivo without invasive procedures, preventing its use in patient assessment.

**Fig. 1:**
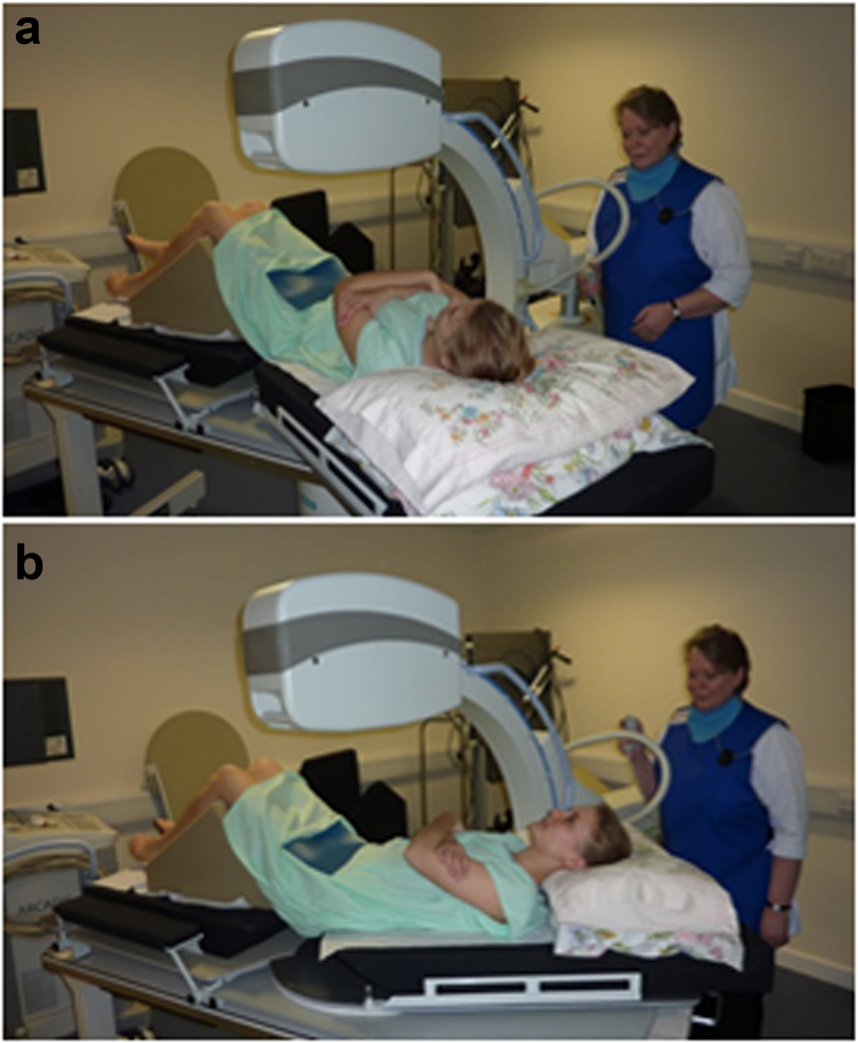
QF passive motion platform: **a** Swung left **b** Swung right

A number of studies have used quantitative fluoroscopy (QF) for the measurement of inter-vertebral motion *in vivo* [11–15]. QF provides continuous inter-vertebral motion information in both flexion-extension and lateral flexion. Patients lie passively on a robotic passive motion platform (Fig. 1a, b) which bends them at a standardised range and velocity while fluoroscopic sequences of inter-vertebral motion are obtained for measurement using image processing codes. This patient orientation minimises muscle activity and allows the intrinsic passive holding element (disc and ligament) restraints to be characterised.

QF has been used *in vivo* to study lumbar intervertebral motion patients and healthy controls [15]. An early version of this technology used weight bearing cineradiography and manual image registration to measure sagittal intervertebral angular motion as trunk motion progressed and claimed to be a surrogate for the NZ [16]. Later studies using fluoroscopy described this parameter as “the slope of the IVFE curve” and “the intervertebral attainment rate” [11, 13, 17].

**Fig. 2:**
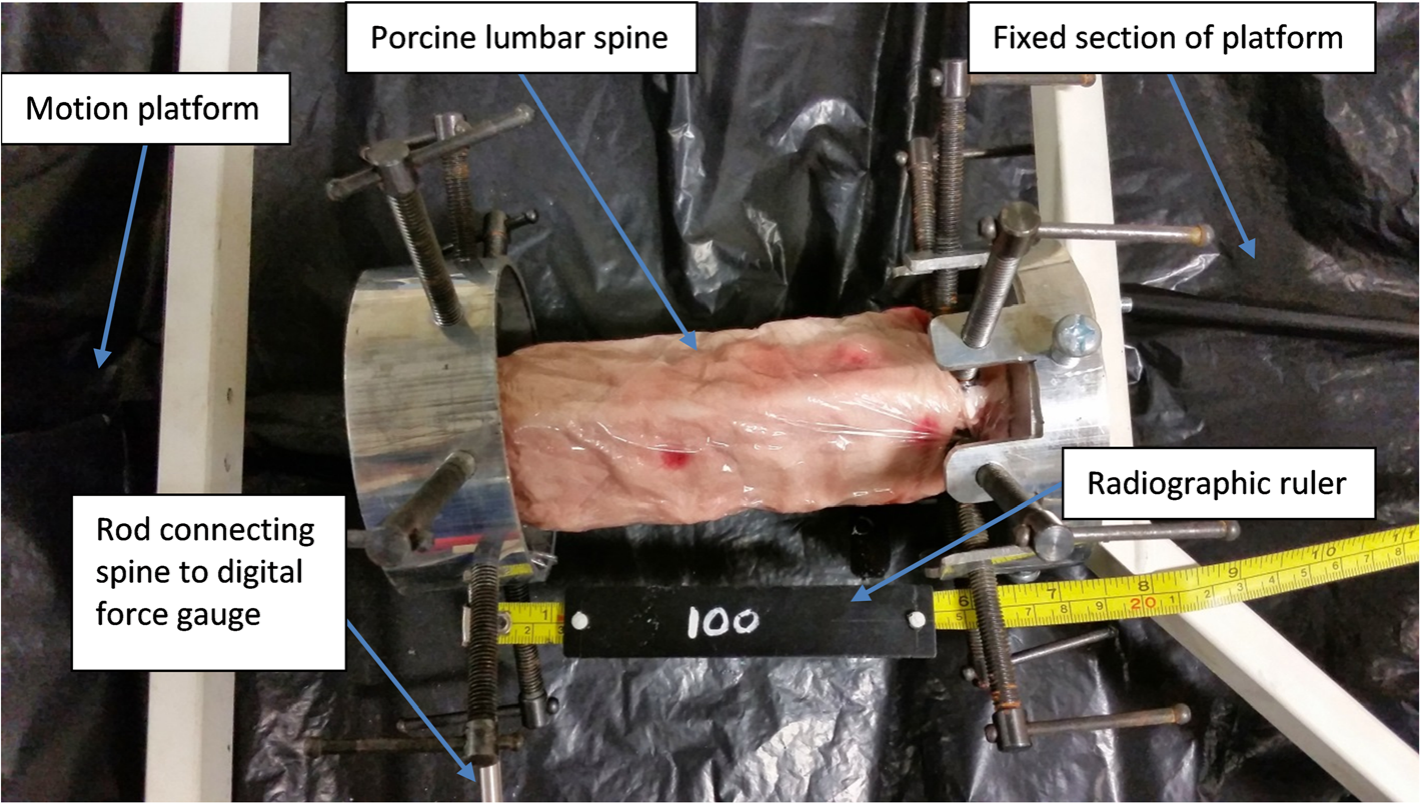
Porcine lumbar spine testing apparatus and motion platform seen from above

Although most studies have concentrated on flexion-extension motion, lateral flexion has also been linked to segmental instability [18, 19]. Furthermore, lateral flexion stability has been shown both to be affected by discectomy and altered in lower limb amputees [20, 21]. Studies by our group used the ratios of the intersegmental bending gradients in the initial 10° of standardised trunk lateral flexion to express the initial attainment rate in an attempt to obtain a more standardised NZ surrogate [15] (Fig. 2).

Both the initial attainment rate and the NZ are expressions of intervertebral laxity. If a relationship is found to exist between them, it would provide evidence of the criterion validity of the former and demonstrate that this *in vivo* assessment of intrinsic lumbar segmental robustness might be used as a relatively non-invasive diagnostic tool in patients with persistent back disability where stability is in question. This study therefore sought to explore a methodology for determining this using a multi-segmented porcine lumbar spine with segments L1 to L5. The bending moments, intervertebral motion and global motion were recorded together using QF, using the same procedures as in lateral flexion QF studies of patients.

## Methods

### Apparatus

A fresh 5-segment porcine lumbar spine (L1 to L5) was prepared as recommended for the biomechanical testing of vertebral specimens [22] The porcine spine is said to have an anatomy that geometrically and biomechanically resembles that of the human spine [23, 24]. The paraspinal muscles were completely excised and all ligamentous components, including the interspinous ligament were preserved [24]. The specimen was preserved wrapped in saline-soaked gauze, covered in cling film and frozen for storage. It was thawed over 12 h before testing, mounted in a horizontal testing frame with the L1 and L5 vertebrae secured by metal halos and circumferential bolts. The same robotic horizontal motion platform used to provide controlled passive motion in patients receiving quantitative fluoroscopy examinations was used for testing (Atlas Clinical Ltd.) (Fig. 2). L1 was attached to the movable segment of the platform and L5 to the fixed segment.

A digital force guage (Omega Engineering Ltd DFG35-10, range 50 N, resolution 0.05 N, sampled at 125Hz) was rigidly connected to the movable part of the motion platform holding the superior vertebral segment. The motion of a connecting rod forced the specimen through a 40° arc, as applied in patient protocols [25], simultaneously transmitting continuous force data from the rod to a laptop computer. The force data were co-ordinated with the digital time stamp output of the motion platform’s motor, which moved the specimen at a uniform velocity of 6° per second at a standardised ramp-up speed over the first second of the motion. This velocity derived from the need to replicate the image recording protocol used in patients, where trade-offs on tolerance, safety and X-ray exposure led to a consensus on these settings [25].

### Data collection

Fluoroscopic sequences of left and right lateral flexion were recorded at 15 frames per second over 15 s using a Siemens Arcadis Avantic fluoroscope VC10A portable C-arm fluoroscope (CE012), whose primary beam was centred on the disc space between the L3 and L4 vertebrae of the specimen. The image field included all 5 segments in all frames so that each vertebra could be tracked and the fluoroscope incorporated automatic distortion correction. Before recording the motion, a calibration image was acquired using a radiographic ruler comprising of two metallic beads of known diameter (4.4 mm) set 100 mm apart into a plastic bar and placed adjacent to the porcine spine and perpendicular to the primary-ray beam in the image field. A single fluoroscopic image was acquired so that this could be used as a scaling factor to calculate the distances between objects in the image sequences.

As in the protocol for patient recordings, the spine was preconditioned by performing four consecutive out and return lateral flexion sequences increasing from 10° up to 40° to replicate this. Ten consecutive recordings were then made of 40° left lateral flexion sequences. The spine was then replaced in a ‘neutral’ position where the force applied by the motion platform was as close to zero as possible. The same procedure was followed for right lateral flexion, however, due to the configuration of the apparatus only a maximum of 30° was achievable for right lateral flexion.

### Image analysis

Outlines of the vertebral body borders of the first image were marked using the computer’s cursor in the first of each sequence of images in a manner identical to the patient mark-up protocol. The positions each of the vertebrae in each of the fluoroscopic images were calculated using automated frame to frame registration codes written in Matlab (The Mathworks Ltd. Cambridge) producing continuous tracking of each vertebral body image throughout the sequences [12]. Trackings were verified visually by a trained operator and the means of the positions of each vertebral section were generated as an output. Average angular motion was smoothed by Tikhonov regularization to reduce inter image variation as with the analysis in living subjects [26, 27].

**Fig. 3:**
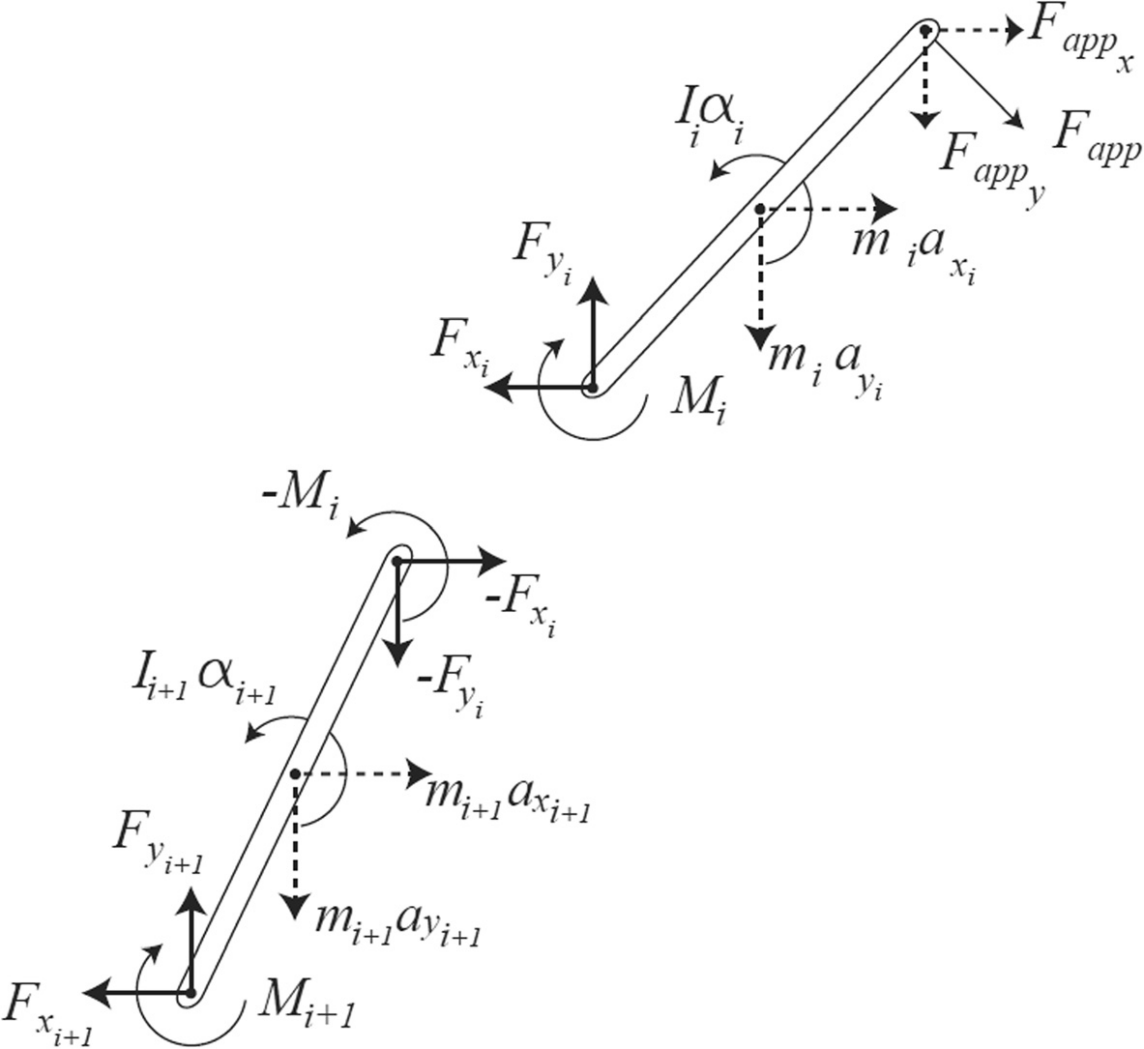
Mechanical model of two successive vertebrae, modelled as having negligible thickness and uniform mass distribution. The figure shows action and reaction forces, net moments of force, and all linear and angular accelerations. Gravitational forces are ignored as they are not applicable in the plane of motion

The changing intervertebral angles of the specimen were co-ordinated with the timing and position of the motion platform. The intervertebral angles of the specimen when the motion platform reached 10°, the moments applied at each intervertebral joint and the motion platform rotation were recorded dynamically. The positions of the point of load application/measurement and the individual joint centres were derived from the trackings of each vertebra in each image frame. Since the centres of rotation between vertebrae are not generally to be found in the joint centre and due to the elasticity of the intervertebral joint, these distances varied slightly during motion and were incorporated into the continuous calculation of moments as detailed below. Forces and moments could not be measured directly at each joint, therefore estimation of forces and moments of forces were derived from the kinematics and inertial properties of the spine by applying the process of inverse dynamics. Modelling the spine as a series of free bending rods of negligible thickness and with uniform mass distribution, an estimation of forces and moments was derived based on D’Alembert’s principle (Fig. 3). One can write the Newton-Euler equations as:

1 $$ {{\displaystyle \sum \mathbf{F}={m}_i\mathbf{a}}}_i\iff \left(-{\mathbf{F}}_{i-1}\right)+{\mathbf{F}}_i+{m}_i\mathbf{g}={m}_i{\mathbf{a}}_i $$

2 $$ {\displaystyle \sum \mathbf{M}={I}_i{\boldsymbol{\upalpha}}_i}\iff \left(-{\mathbf{M}}_{i-1}\right)+{\mathbf{r}}_{i-1}\times \left(-{\mathbf{F}}_{i-1}\right)+{\mathbf{M}}_i+{\mathbf{r}}_i\times {\mathbf{F}}_i={I}_i{\boldsymbol{\upalpha}}_i $$

where **F**_*app*_ is the applied force, **F**_*i*_ is the reaction force, **r**_*i*_ is the distance from the segment centre of mass to **F**_*i*_, since the geometrical centre is considered to be the centre of mass − **r**_*i*_ is the distance from the segment centre of mass to **F**_*app*_, *m*_*i*_ is the mass of segment *i*, g is gravity vector, **α**_*i*_ is the angular acceleration, *Ii* is the moment of inertia and × represents the vector (cross) product. Since gravity is acting perpendicular to the plane of measurement it can be ignored as in Fig. 3.

From Equations 1 and 2 one can calculate each reaction force (**F**_*i*_) and joint moment (**M**_*i*_) acting on each vertebra of the spine.

**Fig. 4:**
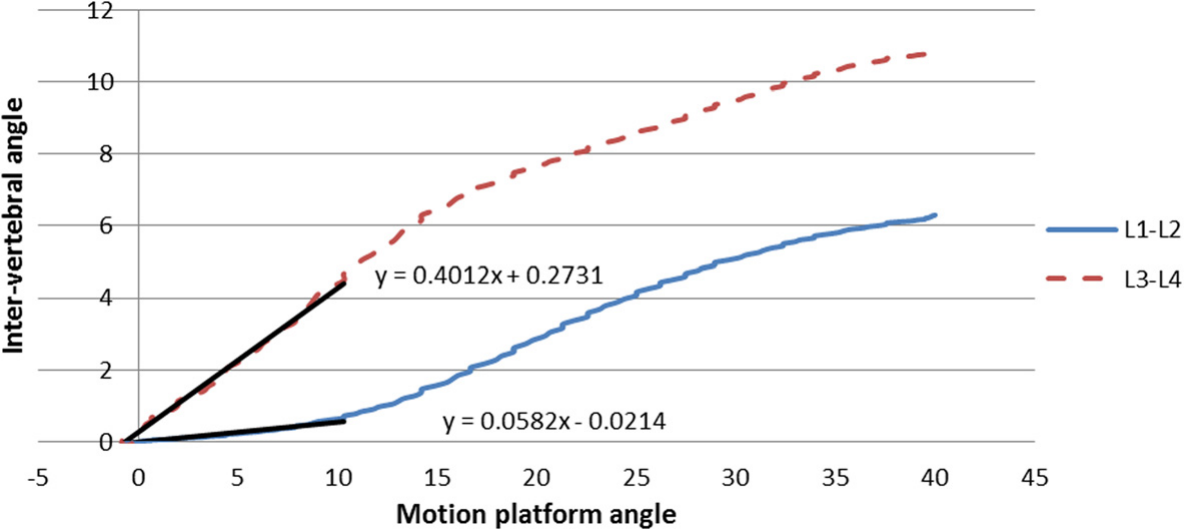
Examples of initial attainment rate calculation: Gradients of inter-vertebral and platform motion in first 10 degrees of platform motion (two intervertebral levels)

Initial attainment rate was calculated as the ratio of the slopes of the first 10° of platform rotation and inter-vertebral rotation over the contemporaneous outward displacement of the latter (Fig. 3). If the motion segment did not rotate by at least 2.5° over this part of the motion (being twice the inter-observer error of the measurement of rotational deformation with this method) the segment was considered stiff and the initial attainment rate was not calculated [25] (Fig. 4).

**Fig. 5:**
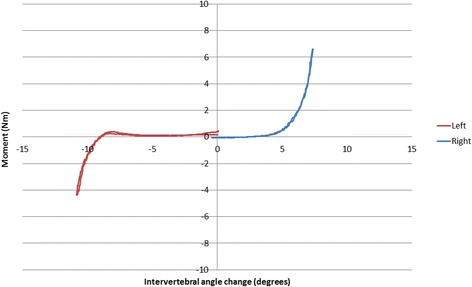
Example of a force deformation curve from an L3-4 motion segment undergoing left and right lateral flexion

The dynamic NZ was taken to be the inter-vertebral angle at the end of the region confined by a slope of +0.05 Nm/degree [28]. Samples of the force-deformation curves for all levels and directions in the specimen were examined to confirm that this was a reasonable assumption for this experiment (Fig. 5).

### Statistical analysis

All data were tested for normality using the Shapiro-Wilk test. The inter-vertebral angle at 10° of platform motion, the dynamic NZs and the initial attainment rates were calculated for each intervertebral level and direction. Correlations between the dynamic NZs and the initial attainment rates in each segment were determined for the pooled data (*n* = 52) and for left and right separately using the Spearman rank correlation coefficient for non-normally distributed data. The cut-off for statistical significance was set at a *P* value of 0.05.

## Results

The mean (SD) (L1-5) ranges of motion for each direction, as measured on the fluoroscopic images were: left 33.5°(1.2) and right 28.3°(0.9), which represented 83 % and 94 % of platform motion respectively. The initial attainment rates for left and right lateral flexion and the pooled data are shown in Table 1.

**Table 1:** Median segmental initial attainment rates for left and right lateral flexion

Left					Right				
	Median	Upper quartile	Lower Quartile	N		Median	Upper quartile	Lower quartile	N
L1-2	-	-	-	0	L1-2	0.204	0.351	0.271	7
L2-3	0.310	0.319	0.302	10	L2-3	0.331	0.342	0.300	10
L3-4	0.406	0.413	0.383	10	L3-4	0.339	0.344	0.333	10
L4-5	-	-	-	0	L4-5	0.239	0.248	0.236	6

The levels of nonparametric correlation between initial attainment rate and dynamic NZ (Fig. 5) were substantial and highly significant for left and combined left-right and moderate for right alone [29] (Table 2).

**Table 2:** Correlations between initial attainment rate and dynamic NZ for pooled levels (L1-2 to L4-5)

	Rho^a^	2-sided p	Number
Left and Right	0.72	0.0001	52
Right	0.55	0.0012	32
Left	0.75	0.0002	20

^a^Spearman’s rank correlation

## Discussion

### Main result

These results are similar to previously published in vivo values for healthy human controls [15] and suggest that there is a relationship between the initial attainment rate and the dynamic NZ. The range of upper quartiles for initial attainment rate (0.204–0.413) were comparable to the upper reference ranges found *in vivo* (0.290–0.429) [15]. However, initial attainment rate and the dynamic NZ are not usually perfectly coincident because they do not measure the same thing; NZ reflects resistance to a pure moment and attainment rate the inter-vertebral motion velocity compared to trunk motion. Furthermore, it is not suggested that the NZ can be calculated from the initial attainment rate, but merely that they are linked in a way that would allow the order of NZ length to be determined from a set of specimens or patients based on initial attainment rate results. In this experiment, they both appear to reflect the intrinsic restraining properties of the inter-vertebral linkages, although the differences need further explanation. In addition, the 10° cut-off used historically to define initial attainment was arbitrary. A better justified calculation may be provided by considering the subsequent work of Smit et al. [30].

### Learning points as an exploratory study

Some of the motion of the frame (40°) was not transferred to the vertebral segments, as 6.5° (left) and 1.5° (right) respectively were lost. This may be due to the use of retaining bolt heads into the bone, calling for a better fixation method. This may have affected the correlations. In addition, two of the segments (L1-2 and L4-5 left) did not reach the required 2.5° required for initial attainment rate to be reported (Table 1). This is likely to be a prevailing feature of multi-segmental examinations, especially if segmental levels are not challenged. Future experimental setups should ensure that equal ranges of the motion platform are obtained.

In calculating the point of inter-vertebral motion from which initial attainment rate measurement begins, fluctuations can occur. If these are prominent, the initial attainment rate value may alter and the method chosen for smoothing to obtain an average value, as well as the ramp-up speed, could affect initial attainment rate values. An international forum on the use of QF suggested this preferred smoothing function but that these values should be kept under review [25–27].

**Fig. 6:**
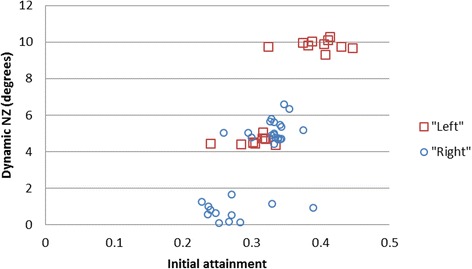
Scatter plot of dynamic NZ (degrees) against initial attainment for left and right lateral flexion

Another question might be why there was not symmetry in the measurement results. The dynamic NZs were generally of a greater order for left lateral flexion, (median left = 7.17°, median right = 4.70°) but over a higher range (range left = 5.90°, range right = 6.49°). This might be the result of lower ranges of right global bending during pre-conditioning and repeated motion and/or alternatively, greater laxity at L3-4 in left lateral flexion (representing the upper cluster in Fig. 6) as a physiological variant. Further studies using multiple specimens and symmetrical testing should clarify this.

### Relevance to clinical studies

In patient and volunteer research studies, the presence of a greater volume of soft tissue between the motion frame and the segment will add noise to the calculation of the initial attainment rate. It might be expected that laxity would be associated with a greater overall range of the segment, but may also be affected by the soft tissue mass. The extent of this might be explored *in vivo* by comparing the initial attainment rates to the overall segmental ranges obtained using QF and to body-mass index.

An additional major challenge in passive system spine kinematics research lies in the complexity of upright motion. This adds the influence of unaccounted variations arising from muscle motor control and body segment mass. However, it also extends the scope of the kind of stability parameters that can be considered.

### Suggestions for further work

The present studies were limited to lateral flexion, where in some circumstances stability may be important. However, the greatest interest in stability, especially for purposes of surgical decisions, focuses on the sagittal plane, where translation is the main kinematic measure used in estimating stability [5]. Studies of the correlation between this and initial attainment rate in the sagittal plane would further inform the use of initial attainment rate in the assessment of patients for segmental laxity.

## Conclusion

The ability to measure inter-vertebral laxity in patients with QF is a step forward in the assessment of chronic back pain where mechanics is thought to be important. This study used the passive recumbent QF protocol in a multi-segmental porcine model for assessing the intrinsic intervertebral responses to a minimal bending moment. It found there to be good correlation between the initial attainment rate and the dynamic NZ, thereby opening the possibility to measure passive system inter-vertebral laxity in clinical studies. However, this was an exploratory study based on repeated measurements in a single specimen, albeit a multilevel one. Therefore, the results, although likely to be important, should be treated with caution. Further, multi-specimen *in vitro* studies are now warranted.
